# NeuroPG: open source software for optical pattern generation and data acquisition

**DOI:** 10.3389/fneng.2015.00001

**Published:** 2015-03-02

**Authors:** Benjamin W. Avants, Daniel B. Murphy, Joel A. Dapello, Jacob T. Robinson

**Affiliations:** ^1^Department of Electrical and Computer Engineering, Rice UniversityHouston, TX, USA; ^2^Hampshire CollegeAmherst, MA, USA; ^3^Department of Bioengineering, Rice UniversityHouston, TX, USA; ^4^Department of Neuroscience, Baylor College of MedicineHouston, TX, USA

**Keywords:** optogenetics, electrophysiology, software, pattern stimulation, imaging, DMD, Polygon400

## Abstract

Patterned illumination using a digital micromirror device (DMD) is a powerful tool for optogenetics. Compared to a scanning laser, DMDs are inexpensive and can easily create complex illumination patterns. Combining these complex spatiotemporal illumination patterns with optogenetics allows DMD-equipped microscopes to probe neural circuits by selectively manipulating the activity of many individual cells or many subcellular regions at the same time. To use DMDs to study neural activity, scientists must develop specialized software to coordinate optical stimulation patterns with the acquisition of electrophysiological and fluorescence data. To meet this growing need we have developed an open source optical pattern generation software for neuroscience—NeuroPG—that combines, DMD control, sample visualization, and data acquisition in one application. Built on a MATLAB platform, NeuroPG can also process, analyze, and visualize data. The software is designed specifically for the Mightex Polygon400; however, as an open source package, NeuroPG can be modified to incorporate any data acquisition, imaging, or illumination equipment that is compatible with MATLAB’s Data Acquisition and Image Acquisition toolboxes.

## Introduction

Optogenetic techniques allow scientists to rapidly manipulate cellular activity by illuminating genetically encoded, light-sensitive proteins (Nagel et al., 2003; Boyden et al., 2005; Zhang et al., 2007; Miesenböck, 2009). Photostimulation of these proteins can affect many cellular behaviors including depolarization or hyperpolarization of the cellular membrane (Boyden et al., 2005; Zhang et al., 2007), gene regulation (Liu et al., 2012a; Motta-Mena et al., 2014), and signaling pathway activity (Airan et al., 2009). One of the main advantages of optogenetics is the ability to modulate the activity of specific subsets of cells. For example, optogenetic techniques can target groups of genetically similar cells using cell-type-specific promoters (Sohal et al., 2009; Cardin et al., 2010; Palmer et al., 2011; Kwan and Dan, 2012), or transgenic animals (Gradinaru et al., 2009; Liu et al., 2012b; Asrican et al., 2013). Even greater specificity is achieved by focusing light to the cell body or sub-cellular structure (Wang et al., 2011; Oron et al., 2012; Smedemark-Margulies and Trapani, 2013; Hochbaum et al., 2014). In this way, scientists can modulate the precise spatiotemporal activity patterns of many individual cells in a localized region with the hope of revealing how information is processed in neural microcircuits (Kwan and Dan, 2012; Hooks et al., 2013; Silasi et al., 2013).

One of the main challenges for using optogenetics to study the roles of individual neurons in a neural network lies in generating the spatiotemporal pattern of illumination necessary to modulate specific combinations of single cells. The three main approaches to activate neurons with cellular resolution include scanning lasers (Wang et al., 2009; Oron et al., 2012; Paz et al., 2013), spatial phase modulation (SPM; Shoham, 2010; Smedemark-Margulies and Trapani, 2013), and digital micromirror devices (DMDs; Leifer et al., 2011; Packer et al., 2013; Smedemark-Margulies and Trapani, 2013; Hochbaum et al., 2014). Each approach has unique advantages. Scanning lasers deliver the entire source power to a small focal spot, making this approach ideal for multiphoton microscopy. Because the focal spot is typically much smaller than the cell body, to efficiently stimulate neural activity the beam is often scanned rapidly over the cell body (Wang et al., 2011; Smedemark-Margulies and Trapani, 2013). However, since the laser can typically only illuminate one location at a time, it is difficult to activate combinations of individual neurons simultaneously. One approach to illuminate many spots simultaneously employs SPM to form multiple focal points for a single laser (Andrasfalvy et al., 2010; Reutsky-Gefen et al., 2013). The main advantage of the SPM is the ability to focus a laser to arbitrary points in 3D; however, the power at each focal point decreases with the number of focal spots, limiting the total number of neurons that can be simultaneously activated (Peron and Svoboda, 2011). As an alternative to laser-based systems, DMDs can simultaneously illuminate hundreds of thousands of points simultaneously (limited by the pixel count of the DMD). Furthermore, because the power is distributed equally to each mirror, the power delivered to each point is independent of the number of points illuminated. The drawbacks of DMDs compared to scanning lasers and phase modulation include higher black levels of illumination (typically some light reaches the sample when the mirrors are switched to the off state), and lower power at each point since each mirror uses only a fraction of the source power. Regardless of these drawbacks, DMDs can effectively stimulate neurons expressing ChR2 (Farah et al., 2007; Zhu et al., 2012) and cost significantly less than laser-based systems. For example, most microscopes can be upgraded to incorporate DMD illumination for less than $10,000. Considering the many advantages of DMD illumination we developed the NeuroPG software suite to streamline DMD-based optogenetic experiments.

The NeuroPG software we developed for DMD-based neural circuit studies combines and synchronizes optical pattern generation with image and electrophysiology data acquisition. While similar open source software exists for laser-scanning systems (Suter et al., 2010), we found no open source solution for automating DMD illumination and neural data acquisition. In an effort to reduce the software development time for labs interested in a low cost method for spatiotemporal manipulation of neural circuit activity, we developed our flexible software platform to combine the control and coordination of both the stimulation and acquisition hardware. As a DMD illumination device we chose the Polygon400 DMD from Mightex since it can be configured with up to three different LEDs and easily adapted to most commercial microscopes.

The NeuroPG software consists of independent software control modules that can be easily modified and expanded. As an example, we have developed several routines to aid with neural circuit mapping and have included these routines in the software package. Specifically we have created tools to define stimulation patterns and to automate simultaneous stimulation and data collection. Once a stimulation protocol is complete, NeuroPG automatically associates the measured responses with the corresponding stimulation region and presents this information graphically, so it may be easily reviewed in real-time. In addition to the graphical output, the raw data and metadata describing the parameters used for the routine are stored in separate files. To aid in subsequent data analysis, NeuroPG includes MatPad, a logging system that records details of the experimental protocols and allows the user to enter and edit notes. Also included is CameraWindow, a MATLAB based camera control suite that can be configured to any camera compatible with MATLAB’s Image Acquisition Toolbox. CameraWindow can run independently or alongside NeuroPG and provides controls and visualization tools useful for cell patching and image acquisition.

## Methods

### Web site

The nueroPG website can be found at https://github.com/RobinsonLab-Rice/NeuroPG. The site includes downloads and documentation for all related software. Any bugs or technical support issues should be reported through the website. Updates and support information will be posted as available.

### Software development

NeuroPG was developed and tested on computers running Windows 7 64-bit and Windows 8 64-bit and should work properly on any such system. Running NeuroPG on 32-bit systems is not recommended. NeuroPG has not been tested on computers running Linux or OSX.

### Hardware and software setup

#### MATLAB

NeuroPG runs in MATLAB (version 2012b and higher; Mathworks, Natick, MA, USA) as a collection of graphical user interfaces (GUIs), classes, and functions. It requires that the Image Acquisition Toolbox be installed for camera functionality. It also requires the Data Acquisition Toolbox for timing, triggering, and data acquisition.

#### Workstation

To run NeuroPG, a computer must have an interface with a data acquisition board (DAQ) either through PCI/PCIe expansion slots or via USB2/3 as required by the DAQ. For imaging, it must also connect to a compatible camera. It is strongly recommended to run a 64-bit version of Windows and have 8 GB or more of high speed RAM. It is also suggested that the workstation have more than one monitor so that image and data acquisition, as well as DMD-control can be viewed simultaneously on the desktop.

#### Data acquisition hardware

Currently, only National Instruments (NI; Austin, TX, USA) DAQ hardware supporting session based control (NI-DAQmx drivers) is supported by NeuroPG. At least three analog input channels and one counter/timer output are required but do not need to be on the same device. NeuroPG was tested using the NI USB-6259 and the NI PCIe-63321 X Series.

#### Amplifier

NueroPG has been tested using Axon Instruments Multiclamp 700B amplifiers but should be compatible with any electrophysiology amplifier with analog output signals that can interface with an NI DAQ module. Scaling of data signals can be done manually within nueroPG so that signal values match actual data values.

#### Camera

NeuroPG, through CameraWindow, supports any camera compatible with the Image Acquisition Toolbox. To control specific camera properties within CameraWindow, the user must generate a configuration file specific to the camera in use. This can be done manually or with the included NeuroPG configuration tool. Example configuration files are available on the website.

#### Digital micromirror device

The Mightex Polygon400 (Mightex, Toronto, Ontario, Canada) is currently the only supported DMD; however, the basic pattern generation and triggering routines are not specific to the Polygon400 hardware. Because NeuroPG accesses the Polygon400 through a MATLAB class any device with a MATLAB control class can be adapted for use with NeuroPG. Creating a MATLAB control class requires access to the device’s software development kit (SDK) and serves as a wrapper that enables MATLAB to interact with the device through its drivers. Because of the differences between DMD systems and their SDKs, we cannot guarantee that all the NeuroPG functions will be compatible with other DMD systems. However NeuroPG can in principle be used with any DMD system that has an SDK by writing similar MATLAB control classes. These control classes will be hosted on the NeuroPG GitHub repository as they become available.

#### Rat hippocampal cell culture

All animal experiments described were carried out in accordance with the National Institutes of Health Guidelines for the Care and Use of Laboratory Animals. Neurons were cultured on an astrocyte feeder layer as described in various protocols, with minor modifications (Brewer et al., 1993; Albuquerque et al., 2009). Both cell types were derived from hippocampal tissue from E18 Sprague Dawley rats that was purchased from BrainBits, LLC. Astrocyte cultures were established by growing cells from dissociated hippocampal tissue in NbAstro medium (BrainBits). Astrocytes were harvested with Tryple (Life Technologies) and plated on PDL-coated 12 mm coverslips at a density of 5,000 cells/mm^2^. E18 hippocampal neurons (BrainBits, LLC) were plated on astrocyte substrate at a density of 10,000 cells/mm^2^ in NbActiv1 medium supplemented with 3% fetal bovine serum. 50% of the medium was replaced with fresh medium every 3–4 days. Experiments described herein were performed at DIV 7–14.

#### Lentiviral transduction

Neuron cultures were transduced with Lenti GFP-ChR2 lentivirus purchased from UNC Vector Core (Chapel Hill, NC). Transductions were performed at 0 DIV at 25 MOI, based on concentration of lentivirus stock provided by UNC Vector Core.

## Results and discussion: description of the software

### Overview

**Figure 1:**
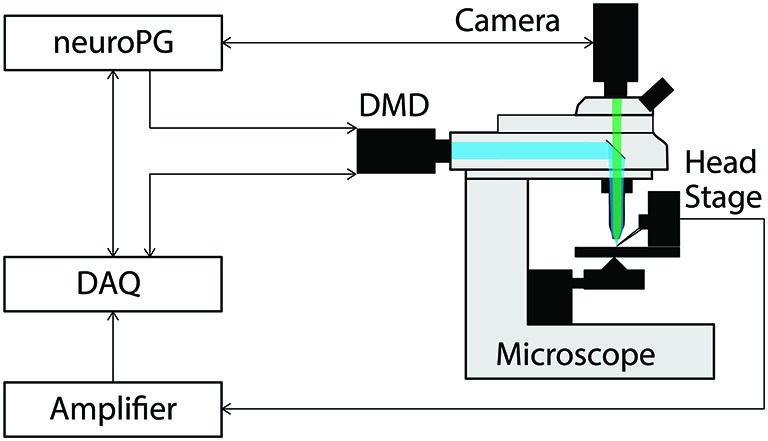
**Connection diagram for the example procedures discussed in this paper**. The DMD output trigger and the signal measured by the head stage are recorded as analog signals by the DAQ to facilitate precise temporal alignment between stimulation and response in the experiment. The DAQ signals the DMD using timed TTL pulses as configured by NeuroPG. All connections with NeuroPG are bi-directional, digital signals. The blue beam represents excitation light reflecting off the DMD and the green beam represents light fluorescently emitted from the sample.

To demonstrate how NeuroPG can be used for neuroscience experiments that require coordinated DMD stimulation and electrical recording, we mapped the response of cultured rat hippocampal neurons to optical stimulus at different locations. The hardware configuration for this experiment is shown in Figure 1. NeuroPG runs on a Dell desktop computer and communicates with a Hamamatsu ORCA-03G camera, an NI USB-6259, two patch clamp headstages, an amplifier, and the Mightex Polygon400 DMD.

For our example experiment, NeuroPG generated grid patterns with increasing resolution, stimulated an E18 rat primary hippocampal neuron transduced with Lenti GFP-ChR2, and generated heat maps based on the magnitude of the resulting depolarization as measured with whole-cell patch clamp electrophysiology. With minor changes to the code or an imported evaluation function, heat maps could alternatively be generated based on hyperpolarization or firing rate. Instructions for generating custom evaluation functions are posted in the documentation available in the NeuroPG GitHub code repository. The experiment is discussed further in Section Example 2: Dendritic Integration and the final heat maps can be seen in Figure 5.

**Figure 2:**
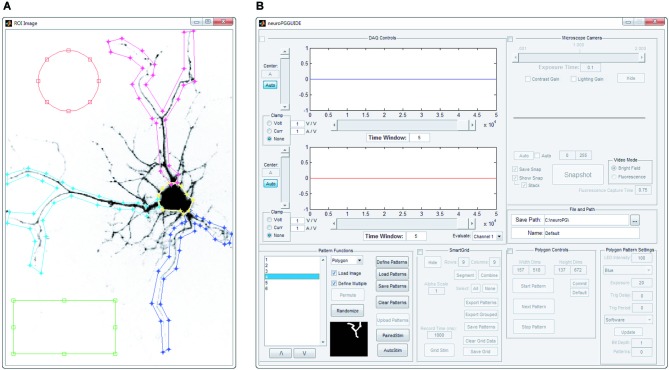
**Manual pattern generation**. ROIs are manually defined as overlays on an image of the field of view that will be stimulated. **(A)** Screenshot of circular, rectangular and spline ROIs, manually defined by drawing on top of the acquired image. **(B)** Screenshot of the main window of NeuroPG, which is updated with the list of ROIs defined in panel **(A)**.

**Figure 3:**
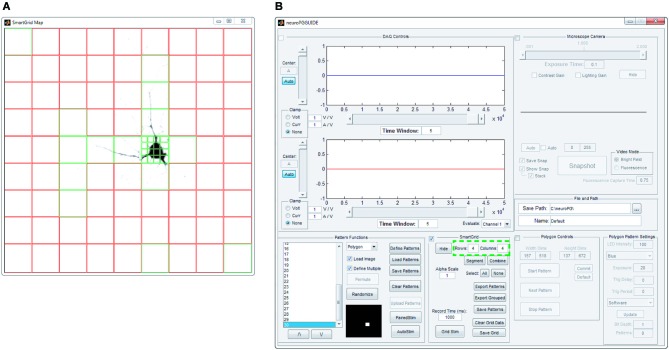
**SmartGrid pattern generation**. A grid of ROIs is generated based on user-defined grid dimensions (number of rows and columns—highlighted in panel **(B)**) and the grid is overlaid on an image of the field of view to be stimulated, as in panel **(A)**. SmartGrid can subdivide any of the selected ROIs into a smaller grid based on user-defined dimensions. Selected regions can be uploaded to the DMD, merged or further subdivided. Selected ROIs appear in green, unselected ROIs appear in red.

**Figure 4:**
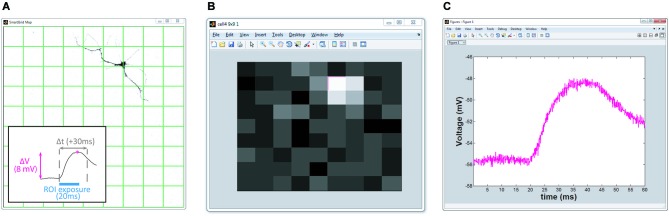
**Real-time HeatMap analysis**. The HeatMap tool visualizes the magnitude of the neuron’s depolarization in response to optical stimulation. Patterns that cause a large depolarization within the stimulation window appear as a “hot spots”. **(A)** Screenshot of SmartGrid being used to generate a grid of patterns. Inset: illustration of how the heat value is calculated. Baseline is calculated from the signal preceding the stimulation period and compared to the maximum depolarization within a user-specified time interval, Δt, following stimulation. **(B)** Screenshot of HeatMap GUI; if the user clicks on an ROI in the heat map (highlighted in magenta) a figure, **(C)**, appears with a plot of the waveform of the selected ROI.

**Figure 5:**
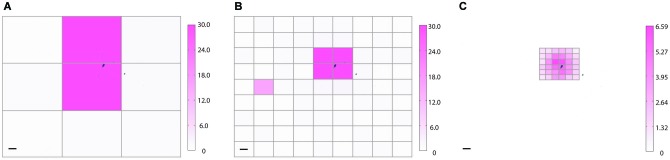
**Coarse and fine heat mapping**. To demonstrate how NeuroPG can perform both coarse and fine sampling of neuronal responses we used the built-in HeatMap function of NeuroPG to map the magnitude of the depolarization resulting from patterned optical stimulation of rat hippocampal neuron expressing ChR2-GFP (not shown) and backfilled with AlexaFluor 594 (black). To prevent action potentials from dwarfing subthreshold depolarization any depolarization greater than 30 mV is plotted as a 30 mV maximum response. Heat maps generated using SmartGrid and HeatMap: **(A)** 3 × 3 grid with each ROI having dimensions of 724μm × 551μm, **(B)** 9 × 9 grid with each ROI having dimensions of 241 μm × 184 μm, and **(C)** a 6 × 6 grid with each ROI having dimensions of 80 μm × 61 μm. Scale bars are 100 μm. Color bar units are in mV.

#### Pattern generation

NeuroPG offers users two methods to define the regions of interest (ROIs) for illumination. Manual pattern generation allows the user to design ROIs of variable size and shape using simple graphic editing tools. Alternatively, patterns can be designed via the SmartGrid tool segments the entire field of view into user-defined rectangles. ROIs are independent of one another and can be composed of any combination or number of DMD pixels. Currently, SmartGrid only generates non-overlapping ROIs but overlapping ROIs can be easily created with the built-in manual ROI generation tool. It is important to note that with most DMD spatial light modulators the optical stimuli will be in focus in the focal plane of the objective lens. To change the depth of the stimulus one can raise or lower the objective lens.

##### Manual generation

Manual pattern generation allows the user to define ROIs as rectangular regions, elliptical regions, or vertex specified polygons. Regions are defined graphically in an intuitive click-and-drag style on top of an image specified by the user. In conjunction with CameraWindow or other microscope imaging software, an image of a sample can be captured and used as a reference for manually creating these regions (Figure 2). Based on the user-defined ROIs NeuroPG then creates pattern masks and uploads these masks to the DMD onboard memory.

##### SmartGrid generation

The SmartGrid pattern generator allows the user to quickly divide the visible area of the microscope into regularly shaped rectangular regions. After loading a background image, the entire field of view is designated as a single region. This region can be divided into a user specified grid where each grid element can be subdivided to the pixel limit of the DMD. Any adjacent regions may also be combined to form a larger single region. Any number of regions in the SmartGrid can be selected and exported as patterns for the DMD. The user can also choose to export selected regions as a grouped pattern, allowing for simultaneous stimulation of non-adjacent areas (Figure 3).

#### Pattern projection and real time analysis

##### Manual mode

NueroPG allows the user to set Polygon400 parameters, load patterns, start or stop pattern sequences, and trigger the next pattern manually all within the GUI. The user can also manually start and stop data recording. Manual control gives the user the most flexibility in controlling the parameters of the experiment. However, because the parameters can be varied throughout an experiment NeuroPG does not currently have any automated routines for analyzing or plotting data recorded during manual control.

##### AutoStim mode

Once a user has defined at least one stimulation pattern, the AutoStim function can automate the process of stimulating the sample and acquiring data. AutoStim initializes the DMD’s settings and places it into external trigger mode. AutoStim then uploads the stimulation patterns in the list and coordinates triggering and data acquisition. Timing for the routine is controlled by the real-time clock in the DAQ board to ensure accuracy and precision. Once the stimulation routine has finished, AutoStim saves parameters, patterns, and recorded data to a user-specified file. AutoStim automatically associates sections of the electrophysiological data recorded during the stimulation routine with their respective stimulation patterns based on the output trigger of the DMD. The stimulation patterns and associated data can be then passed to the HeatMap visualization tool. In AutoStim mode NeuroPG generates a TTL signal that precedes the illumination of each ROI. This TTL signal triggers the DMD and can be split and routed to other systems such as pClamp or automated stages to trigger other events related to the stimulus. The time between the trigger signal and the beginning of stimulation can be adjusted with the Trig Delay property of the DMD to allow for external systems to prepare for each stimulus.

##### PairedStim mode

The PairedStim routine facilitates the investigation of time-dependent processes by illuminating two patterns with a specified delay between exposures. To initialize PairedStim, the user specifies two stimulation patterns and the latency between the stimuli. The two patterns can be identical, independent, or partially overlapping. PairedStim then generates the necessary patterns and settings for the stimulation and uploads this data to the DMD. After PairedStim has executed the routine the recorded data is automatically analyzed and plotted.

##### HeatMap

Included in NeuroPG is a tool that associates collected data with the corresponding stimulation region, evaluates the data, and presents it graphically as a heat map. The default evaluation function plots the maximum depolarization following stimulation. This function identifies the stimulation periods in the data using the DMD output trigger signal and then calculates the difference between the maximum membrane potential within a user-specified time window following stimulation and the average membrane potential prior to stimulation (Figure 4). The regions are mapped onto an area corresponding to the field of view and the alpha values (or color intensity values) of each region are set according to the determined “heat” value. Right clicking any region will open a plot of the electrophysiology waveforms used to calculate the “heat” for that particular region. The function used to evaluate the “response magnitude” can easily be changed as described in Customizing Evaluation Functions. Heat maps that are saved as MATLAB figure files will retain all data and functionality when opened by any instance of MATLAB that has access to the HeatMapBDF function.

### Example 1: heat map generation

One of the advantages of DMD illumination is simple control of the illumination spot size and position. As an example, we used NeuroPG to measure and plot the magnitude of the depolarization as a function of illumination position using a coarse and fine grid size. Adaptive sampling based on the ability to increase the sampling resolution may help to efficiently identify ROIs that have specific influence on neural cells or circuits. To demonstrate our ability to generate heat maps at different resolutions, we used dissociated hippocampal neurons from E18 Sprague Dawley rats, transduced with Lentivirus-ChR2-GFP-CamKII (Boyden et al., 2005) and cultured on hippocampal astrocytes from E18 Sprague Dawley rats. We located a GFP positive neuron using fluorescence microscopy and recorded the transmembrane potential using the whole-cell patch clamp method in current clamp mode. Using SmartGrid, we then divided the field of view into regular rectangular patterns that were exported to the Pattern List and randomized. We then used AutoStim to stimulate each of the ROIs in a randomized order and collect the corresponding electrophysiology data. Once the stimulation was complete, AutoStim divided the recording into sections associated with each ROI and exported the data to HeatMap. To remove any artifacts related to spontaneous activity, we used a simple algorithm in HeatMap to scan for regions that contained spontaneous action potentials that overlapped periods of stimulation and discarded those data. As described in the previous section, HeatMap’s default evaluation function calculated the local baseline membrane potential and determined the “heat” value based on the maximum depolarization relative to this local baseline. The function then generated a figure from this data by coloring each pattern’s area according to the heat value (Figure 5). Selecting any ROI in this figure generates a plot of the data used to calculate that region’s heat value. This interactive feature allows the user to easily examine the waveform of specific ROIs. Over the course of this experiment, NeuroPG automatically saved the raw recorded data, the patterns used for the stimulation, the parameters used in all the evaluation functions, an image of the HeatMap figure, and the interactive MATLAB figure itself.

### Example 2: dendritic integration

**Figure 6:**
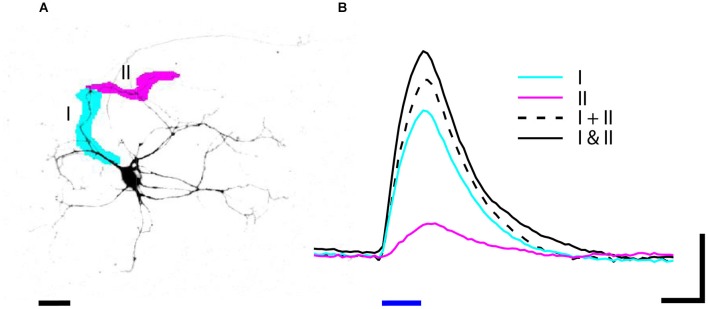
**(A)** Neuron expressing ChR2-GFP was stimulated by blue light (470 nm) illumination with patterns I (magenta) and II (cyan). **(B)** The depolarization in response to 20 ms (blue bar) stimulation was recorded by whole cell patch-clamp electrophysiology. The depolarization in response to stimulating two ROIs simultaneously (solid black I and II) is greater than the summed depolarization of I and II stimulated individually (dashed black I + II). Data is averaged from three trials; scale bars: **(A)** 25 um, **(B)** 4 mV and 20 ms.

Another advantage of DMD illumination is the ability to simultaneously illuminate multiple arbitrarily shaped ROIs. For example, stimulating two regions within the dendritic arbor can reveal how those currents sum at the cell body. To demonstrate how NeuroPG can be used to study dendritic integration we used dissociated E18 rat hippocampal neurons transduced with the Lenti-ChR2-GFP-CaMKII as discussed above. Using manual ROI generation, we targeted two non-overlapping segments of a single dendritic branch. We then stimulated each ROI both individually and together and measured the resulting depolarization at the cell body using whole cell patch-clamp electrophysiology. The results of these measurements are shown in Figure 6. In this case, we found that stimulating the two ROIs together resulted in a depolarization that was larger than the summed depolarization resulting from individual ROI stimulation. This ability to simultaneously illuminate complex spatial patterns makes NeuroPG well suited to study the electrical properties of dendrites and dendritic integration.

### Customizing evaluation functions

We have written the NeuroPG data processing and evaluation functions to allow easy modification and support a variety of experiments. In particular the HeatMap tool is designed to allow users to create as many of their own evaluation functions as they would like. These functions must follow a general template but may have variable numbers and types of input parameters. The template specifies the structure of input parameters and the way in which HeatMap queries the function to learn what parameters need to be passed. An example function using the template is included with the NeuroPG package to facilitate customization. In this way, data collected from an experiment may be processed and visualized using multiple, customizable evaluation methods. For example, the heat maps may be based on the maximum depolarization, latency, or rise time. While the default visualization is a heat map, this visualization can be easily modified by the user to best represent their data.

### Peripheral functionality

#### CameraWindow

CameraWindow is a microscope camera visualization and control utility designed to control any camera compatible with the Image Acquisition Toolbox. It was designed with cell patching in mind and includes a marker that can be repositioned in the camera window by simply right clicking the desired position. It also has digital 2x and 4x zoom that centers the image wherever the mouse is clicked. CameraWindow provides a histogram of image intensity and allows for auto or manual scaling of image intensity in both grayscale and color modes. The user can easily switch between viewing a sample in bright field and in fluorescence by clicking a radio button. Fluorescence mode changes the exposure and contrast settings to user specified values to enhance low light visualization. Changing back to bright field mode restores the settings to their previous state. The Snapshot functionality allows the user to capture images in one of two ways. In brightfield mode, the raw image data is captured using the current settings of the camera. In fluorescent mode, CameraWindow uses a short exposure time to display the live camera image, but switches to a longer exposure time to capture the raw image data. This allows the user to view their sample at a higher frame rate during focusing and positioning and then capture images with longer exposure times. In either mode, the image data is written to a tif file that is named by appending an incrementing number to the specified file name. The user can also display captured images in one of two ways. New images can be displayed by either creating a new window for each image or stacked on top one another in the same window. Stacked images can be cycled through by mouse click and can be used as time-lapse videos or for comparing bright field and fluorescence images. Stacked images are still saved as individual tif files, not tif stacks. CameraWindow also has a video capture function. When the video button is clicked, video frames are captured from the visualization window and stored in a buffer until system RAM is 90% full or the video button is clicked again. The captured frames are then saved to disk as uncompressed AVI files named with the specified name. CameraWindow tracks the amount of available system RAM and calculates approximately how many seconds of video may be captured at any point in time. Any number of camera properties can be adjusted from the CameraWindow control window. The number of camera properties and order with which they appear is specified in the configuration file. The configuration file can be edited manually or through the included configuration functions. The exposure and contrast properties will always appear at the beginning of the property list if they are defined for the camera.

#### MatPad

MatPad is a logging and note utility that NeuroPG uses to log events. MatPad logs changes to NeuroPG settings, as well as the parameters for AutoStim, PairedStim, and all pattern generation methods. MatPad also allows the user to enter their own notes in the log, remove lines from the log, or edit the text of the log directly. The log file name and path are user specified and can be saved in the configuration file or changed manually. If MatPad is directed to use a log file that already exists, it will append new entries into the file without changing any previous data. MatPad is designed to minimizes data loss due to unexpected crashes, shutdowns, or failures by regularly saving the log file to disk. Log files are saved as txt files and can be viewed or edited in any text editor.

### Installation and set up

The current version of NeuroPG along with download and installation instructions can be found on the NeuroPG website. It is distributed as open-source software under the GNU General Public License version 3.0 (GPL-3.0).

Software is included with NeuroPG that will automatically generate the configuration file required for each workstation. It is comprised of four configuration utilities that allow the user to specify default settings and parameters. The utilities for configuring DAQ hardware and cameras will auto-detect available hardware and settings for devices present on the workstation and allow the user to customize how these devices are utilized. All NeuroPG settings are stored in a single file that may be edited manually by the user. Settings are stored in MATLAB mat file format to allow for easy access and editing. Detailed instructions for editing the file manually are included in the documentation provided on the website.

## Software architecture discussion for developers

The NeuroPG platform is modular and uses an object oriented programming approach. Each interfaced piece of hardware is realized within the software as its own object, allowing it to be accessed and controlled by multiple modules without conflict. This means that MATLAB functions which are not native to the NeuroPG platform can share the system’s resources, allowing for customized expansion of NeuroPG’s functionality. NeuroPG is also designed to be event driven. This allows multiple tasks to run in tandem without blocking thereby enabling the user to run their own functions or operations while NeuroPG is active. The event driven design also allows NeuroPG to adjust camera settings or record video while patterns are being stimulated and data is recorded. The extent to which tasks may be run in parallel is impacted by the hardware capability of the computer and resource intensive operations like saving large amounts of data to disk. Because of this, timing and signaling are handled by the DAQ hardware, ensuring precision for both pattern stimulation and data recording. Memory management is carefully handled within NeuroPG using a combination of shared data structures, persistent variables, and class properties. The approach for memory management is designed to reduce redundant data and prevent unnecessary copying or relocation, and is built around MATLAB’s memory management system. Data processing takes advantage of MATLAB’s optimized vector mathematics algorithms to minimize processing time and memory usage. Data recorded during a stimulation routine is kept in pre-allocated buffers within system memory and written to disk only once the routine is complete. These techniques make NeuroPG robust and reliable, with the primary goal of ensuring the accuracy and reliability of experimental data.

## Conclusion

The flexibility, speed and usability built in to our NeuroPG software package in combination with any DMD-equipped microscope provides a powerful platform to support experiments using any variety of proteins from the optogenetic toolkit (Smedemark-Margulies and Trapani, 2013). Specifically the real-time data analysis and visualization as well as flexible stimulation routines allow NeuroPG to improve experimental efficiency for many optogenetic experiments. NeuroPG is currently configured to record two analog electrical signals but could be modified to accept more input channels. The output from any device that can be encoded as an analog voltage signal (e.g., MultiElectrode Arrays, photodetectors collecting bulk fluorescence) could take the place of patch clamp electrophysiology in our example implementation of NeuroPG. While we have discussed neural circuit studies and dendritic integration as potential applications, because NeuroPG can be easily modified to support a vast array of applications, we see this software toolkit as a key building block for the general optogenetic community.

## Conflict of interest statement

The authors declare that the research was conducted in the absence of any commercial or financial relationships that could be construed as a potential conflict of interest.
